# Roles of Cholecystokinin in the Nutritional Continuum. Physiology and Potential Therapeutics

**DOI:** 10.3389/fendo.2021.684656

**Published:** 2021-06-02

**Authors:** Laurence J. Miller, Kaleeckal G. Harikumar, Denise Wootten, Patrick M. Sexton

**Affiliations:** ^1^ Department of Molecular Pharmacology and Experimental Therapeutics, Mayo Clinic, Scottsdale, AZ, United States; ^2^ Drug Discovery Biology theme, Monash Institute for Pharmaceutical Sciences, Monash University, Parkville, VIC, Australia

**Keywords:** cholecystokinin, type 1 cholecystokinin receptor, obesity, appetite regulation, positive allosteric modulator, biased agonist

## Abstract

Cholecystokinin is a gastrointestinal peptide hormone with important roles in metabolic physiology and the maintenance of normal nutritional status, as well as potential roles in the prevention and management of obesity, currently one of the dominant causes of direct or indirect morbidity and mortality. In this review, we discuss the roles of this hormone and its receptors in maintaining nutritional homeostasis, with a particular focus on appetite control. Targeting this action led to the development of full agonists of the type 1 cholecystokinin receptor that have so far failed in clinical trials for obesity. The possible reasons for clinical failure are discussed, along with alternative pharmacologic strategies to target this receptor for prevention and management of obesity, including development of biased agonists and allosteric modulators. Cellular cholesterol is a natural modulator of the type 1 cholecystokinin receptor, with elevated levels disrupting normal stimulus-activity coupling. The molecular basis for this is discussed, along with strategies to overcome this challenge with a corrective positive allosteric modulator. There remains substantial scope for development of drugs to target the type 1 cholecystokinin receptor with these new pharmacologic strategies and such drugs may provide new approaches for treatment of obesity.

## Introduction

Nutritional status reflects the health of an organism, with both extremes of this continuum that represent malnutrition and obesity indicative of and/or causing major health problems. In particular, obesity has become one of the dominant health problems present throughout the world. This has many associated co-morbidities, such as diabetes mellitus and cardiovascular disease, which are responsible for immense expense, suffering, and even mortality. Many potential therapeutic targets have been identified for the treatment of this problem (1, 2). The focus of this review is the role played by one specific gastrointestinal hormone, cholecystokinin, in the physiology of nutrition and potentially for therapeutic approaches to prevent and manage obesity.

The gastrointestinal tract plays a central role as the portal for the nourishment of an organism. The gastrointestinal endocrine system is key for the regulation and integration of multiple processes that contribute to normal nutrient digestion and absorption. This includes the control of secretion of acid and pepsin in the stomach, rate of gastric emptying, biliary secretion and gallbladder contraction, pancreatic exocrine secretion, and intestinal and colonic transit. The gastrointestinal endocrine system provides remarkable orchestration of a complex series of events that contribute to optimal rates of delivery of nutrient-containing chyme having composition ideal for intestinal absorption. Perhaps even more remarkable is the frequency that these processes occur seamlessly in the background, without people being aware of the component events, even after tremendous variation in the size and composition of meals ingested.

## Cholecystokinin and Its Receptors

One of the classical gastrointestinal hormones known to play a central role in regulating nutritional homeostasis is cholecystokinin (3). This is a peptide hormone synthesized and secreted from neuroendocrine I cells scattered throughout the mucosa in the proximal two-thirds of the small bowel. Major stimulants of its release include lipids and protein in the meal. Earliest recognized physiologic effects of this hormone include the stimulation of gallbladder contraction (described as cholecystokinin) and pancreatic exocrine secretion (described as pancreozymin). Ultimately, it was appreciated that the same peptide was responsible for both activities, and what became known as cholecystokinin-pancreozymin, was ultimately shortened to cholecystokinin (CCK). With bile playing a key role in micelle formation for the digestion of fat and pancreatic enzymes critical for the digestion of fat and protein, the rationale for the parallel roles for CCK in this servomechanism becomes clear. Other physiologic actions of this hormone include slowing gastric emptying, to titrate the rate of delivery of nutrients to allow optimal digestion and absorption. All of these actions are important to facilitate nutrient assimilation for the organism.

In modern society, we often have ready access to high caloric foods, and we do not have to expend the energy previously required by our ancestors to gather this food. This has shifted the energy balance, thereby contributing to the tendency for people to be overweight or frankly obese. Teleologically, in attempt to address this, cholecystokinin can also suppress appetite, limiting overeating and weight gain. Indeed, cholecystokinin was one of the first GI hormones recognized as having this effect (4).

The effect of cholecystokinin on appetite is mediated by a class A G protein-coupled receptor (GPCR) identified as the type 1 cholecystokinin receptor (CCK1R) (4). These receptors are present on vagal afferent neurons present within the upper gut wall (5). Those neurons subsequently affect central nervous system nuclei involved in appetite regulation. It is important to remember that the dominant target for controlling appetite is a peripheral CCK1R, and there is no need for a potential drug that targets these receptors to cross the blood-brain barrier. Cholecystokinin also binds with high affinity and acts with high potency at a structurally-related receptor identified as the type 2 cholecystokinin receptor (CCK2R) (6, 7). CCK2R is present on the parietal cell where it mediates gastric acid secretion and on various neurons in the periphery and central nervous system (7). While both CCK1R and CCK2R bind the same peptide with high affinity and signal similarly, they have different ligand recognition properties (6, 7). The CCK2R is also known as the gastrin receptor, with cholecystokinin and gastrin sharing the same carboxyl-terminal pentapeptide amide (GWMDF-amide). Indeed, the peptide pharmacophore for the CCK2R is the carboxyl-terminal tetrapeptide amide (WMDF-amide) that is present in both CCK and gastrin. The pharmacophore for CCK1R extends beyond this small focused region, requiring seven amino acids for receptor activation, including a tyrosine sulfate present uniquely at the position seven residues from the carboxyl terminus of cholecystokinin [(Y-sulfate)MGWMDF-amide].

## CCK1R-Active Drugs for Obesity

When the prevalence and adverse implications of obesity began to be appreciated, cholecystokinin agonists that are active at CCK1R were widely sought as possible therapeutic tools. Indeed, several major pharmaceutical companies launched programs to develop full agonists with cholecystokinin-like activity (8–11). All such agents, including peptides, peptoids and small molecules, have been active in reducing meal size and causing weight loss. Most of the drug candidates in development exhibited selectivity for CCK1R over CCK2R, but one of these candidates exhibited agonist activity at CCK1R and antagonist activity at CCK2R (8). There may have been an advantage of such a compound to not only reduce appetite, but also to reduce acid secretion. However, for approval for an obesity drug, the Food and Drug Administration requires such agents to be more efficacious than acute dieting. No such agents have yet met this primary endpoint in clinical trials.

A challenge in developing effective CCK1R agonists for the treatment of obesity has been side effects of highly potent and/or long-acting full agonists that can cause nausea, abdominal cramping, and diarrhea, with the theoretical possibility observed in rodents of trophic effects on receptor-bearing cells and the possible progression and/or development of pancreatic cancer (11). With the disappointing effects observed in the early clinical trials for obesity and these theoretical side effects and toxicities, the enthusiasm for developing agents that are exclusive cholecystokinin agonists for obesity has waned, and we are not aware of any active drug development programs continuing to pursue this (12). However, CCK1R continues to be a potential target of interest for programs that combine different agents to take advantage of complementary and synergistic effects of distinct hormonal systems (2, 13–15).

Gastrointestinal endocrinology is a field that has progressed through various stages, as described by Jens Rehfeld (16–18). In the early years, many gastrointestinal hormones were discovered based on regulatory activities such as those described above. Subsequently, we have been able to biochemically characterize these hormones and quantify them. This led to increased understanding of physiologic roles, as well as pathologic and even potential therapeutic roles for these hormones. In more recent years, the focus shifted toward the receptors for these hormones. This provided potential targets for drug discovery. Of these receptors, as noted above, even when a hormone has an ideal physiologic effect that could be mimicked by such a drug, there can still be shortcomings and limitations of such agents.

**Figure 1:**
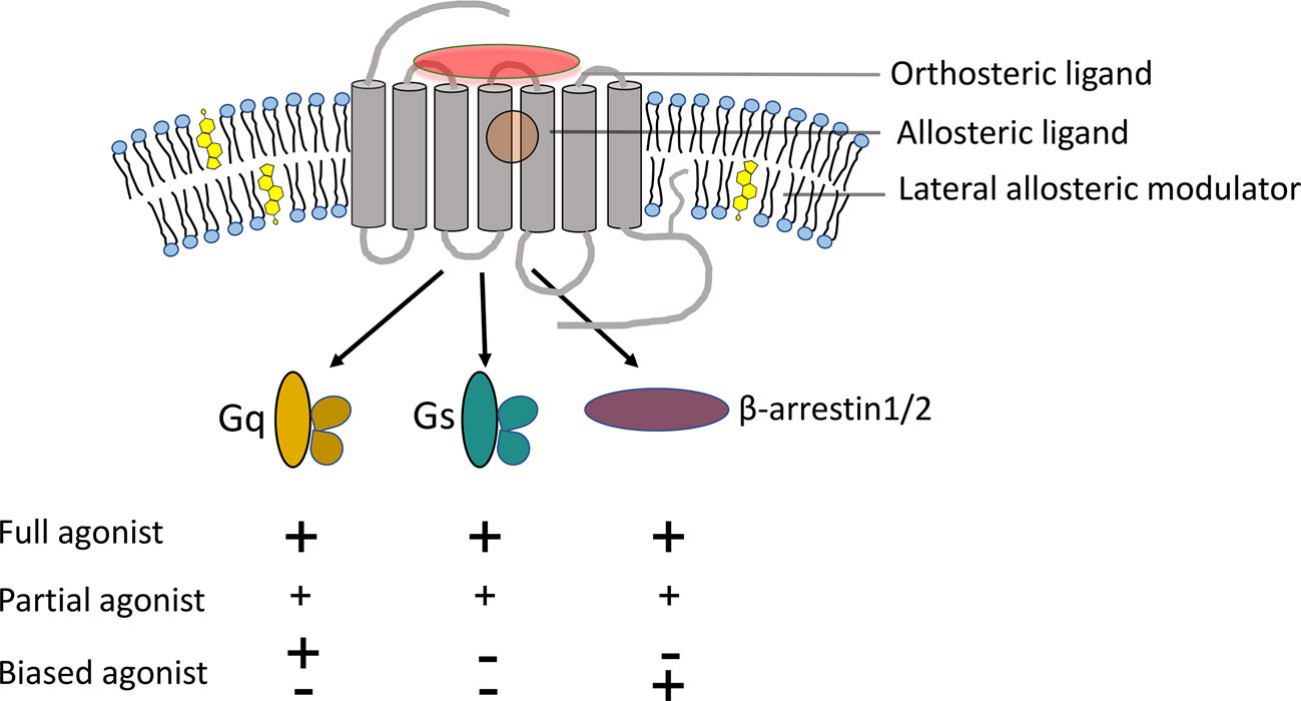
Potential for new types of CCK1R-active drugs. Shown is a diagram of a GPCR, highlighting the orthosteric site of action of the natural agonist ligand and distinct allosteric sites of action for allosteric modulators. These can be lateral allosteric modulation through membrane lipids like cholesterol or through association with other membrane proteins, or *via* ligands that bind to the receptor in places outside of the orthosteric site. Any of these ligands can act as full agonists, stimulating the full range of pleiotropic signaling events activated by the natural agonist, or reduced responses of some of these events (partial agonist and biased agonists). Traditional drug development has focused on full agonists and antagonists (inverse agonists). It is now clear that “texture” in responses can be evoked by allosteric modulation and/or biased agonists.

Molecular pharmacology has recently recognized the potential power of new types of receptor-active drugs providing “texture” to the response, rather than simply acting to fully turn on or off the post-receptor signaling mechanisms (19, 20). This can take the form of biased agonists or allosteric modulators of natural hormonal action (**Figure 1**). We believe that both of these newer types of drugs have the potential to reinvigorate drug discovery efforts targeting the cholecystokinin receptor for the treatment of obesity.

## Rationale for Developing Biased Agonists of CCK1R

After the initial recognition of GPCRs as key conduits for regulation of cellular function, the focus was on signaling through association with a dominant heterotrimeric G protein, as the presumed driver of functional response for this class of receptors. This focus was on signaling mediated by the activated GTP-bound G protein alpha subunit. It was subsequently recognized that the beta-gamma subunits of the G protein could independently mediate signaling activities (21). We now recognize that multiple distinct G proteins can couple with an individual GPCR, and that even non-G protein-mediated signaling can occur (22, 23). For example, arrestin proteins were identified as prominent proximal scaffolds of alternate transducer proteins for activated GPCRs (22). All this contributed to our current concept of pleiotropic signaling initiated by GPCRs. With this came the recognition that various ligands, both naturally occurring hormones and drugs, can stimulate various parts of this signaling machinery in a selective manner (19, 20). Thus, there has been elevated interest in the concept of “texture” of the biological responses.

Almost all of our knowledge of signaling events initiated by cholecystokinin occupation of CCK1R has come from use of synthetic CCK-8 and with a particular focus on intracellular calcium or inositol phosphate as second messengers generated downstream of receptor association with Gq/11 proteins. Although it is now recognized that a variety of signaling events distal to activation of the CCK1R can occur beyond those mediated by Gq/11 proteins (24), essentially all drug discovery activity to date has focused on efforts to identify full agonists by measuring Gq-mediated responses. There are no published data systematically evaluating the broad spectrum of signaling responses to various CCK ligands. Due to the paucity of such data, it is not yet possible to identify the optimal signaling profile for a biased CCK1R-active drug for the management of obesity.

There are at least two lines of evidence pointing toward the potential importance of the broader spectrum of signaling responses initiated at the CCK1R. One of these relates to heterogeneity of responses to two naturally-occurring molecular forms of this hormone, CCK-8 and CCK-58 (25, 26). The other relates to analogues of CCK that have been prepared and used in the laboratory, the most interesting being what has been called a mixed high affinity agonist/low affinity antagonist of CCK1R, JMV-180 [also known as an O-phenyl ethyl ester analogue of CCK, CCK-OPE (27)] (28).

With the pharmacophore of cholecystokinin that is recognized at CCK1R being defined as the carboxyl-terminal heptapeptide-amide, present within synthetic CCK-8, and with this peptide widely available for a very reasonable price, almost all *in vitro* and *in vivo* studies of cholecystokinin biologic activity have utilized this form of this hormone. This includes the feeding studies that have universally demonstrated impact to reduce meal size (4). The physiologic importance of the CCK1R has also been demonstrated with a selective antagonist that has been shown to stimulate food intake (29). However, at least a subset of the studies with CCK-8 also demonstrated shortened interval between meals, offsetting the reduced caloric intake at a single meal; this has reduced the enthusiasm for CCK agonists as satiety agents for the treatment of obesity. However, of particular interest, the physiologic form of this hormone that is believed to be dominant in the circulation is CCK-58 (30). Because this peptide is not readily commercially available and is much more expensive to produce than CCK-8, far fewer studies have been performed with the physiologic peptide. There are now compelling data demonstrating that this form of cholecystokinin not only reduces meal size, but also extends the inter-meal interval (25, 26). Thus, the impact of endogenous cholecystokinin released after eating is a net reduction in caloric intake. The potential differences in signaling events stimulated by these two forms of cholecystokinin that might underly their different physiologic profiles have not been investigated to date.

An analogue of cholecystokinin in which the carboxyl terminus of this hormone was modified to an O-phenyl ethyl ester rather than a phenylalanine-amide linked through a peptide bond was reported to have interesting biological properties. Extensive work of Gardner led to this analogue being described as a mixed high affinity receptor agonist and low affinity receptor antagonist, since it mimicked the increase in pancreatic secretion stimulated by CCK-8, while not exhibiting the supramaximal inhibition of secretion typical of CCK-8 (28). This was also reflected in biological differences in animal models, where infusions of high doses of full CCK-like agonists such as caerulein produced pancreatitis, whereas the analogue peptide did not (31). Moreover, in feeding studies, where full CCK-like agonists reduced meal size, this peptide did not (32). In cellular assays, the peptide analogue appears to elicit a smaller intracellular calcium response than CCK-8 (33), but detailed analyses of the full spectrum of signaling and regulatory events that occur at the CCK1R have not been reported. Rather than representing a mixed agonist/antagonist or even a partial agonist, this peptide is most likely a biased orthosteric agonist. Careful pharmacologic analysis will be important for understanding the translational relationship between distinct modes of cellular signaling and the physiologic effects mediated by the CCK1R.

Based on these existing literature observations, it is likely that some type of biased agonism at CCK1R may have therapeutic benefit. Such bias can be achieved either by orthosteric or allosteric agents with intrinsic biological activity, or could be achieved by pure allosteric modulators that have no intrinsic activity.

## Rationale for Developing Allosteric Modulators of CCK1R

Allosteric modulators interact with receptors at sites spatially distinct from the locus where natural agonist hormones act, termed the orthosteric site (19). This allows such modulators to bind to the receptor simultaneously with natural hormone. Allosteric modulators can sculpt the normal responses to natural hormones, altering the profile of cellular response, while maintaining the physiologic timing of receptor stimulation. The modulatory role of such agents can affect hormone binding and/or biological responses, with the latter including potential for enhancement or reduction of coupling events that can be specific to particular signaling pathways. The allosteric modulatory effects are typically saturable, adding an additional level of safety to the effects of these agents. It has previously been suggested that pure allosteric modulators may be advantageous as CCK1R-active drugs to be used in obesity (34) - a positive allosteric modulator of CCK1R possessing no intrinsic agonist activity could enhance the satiety effect of natural CCK when it is released after a meal (34). The short half-life of circulating CCK peptides would limit the potential for side-effects relating to prolonged receptor activation, adding to the safety profile of such agents.

Extensive work has been done to determine how cholecystokinin binds to its receptor. Like most peptide hormones binding to a GPCR, this peptide interacts primarily at the receptor region sited within external surface of the lipid bilayer, including interactions with extracellular loop regions (35). There is also indirect data suggesting peptide interaction deeper within the helical bundle of this receptor (36). The peptide binding site is by definition the orthosteric ligand-binding site of this receptor. The most direct evidence for the mode of docking this peptide under normal conditions comes from intrinsic photoaffinity labeling studies in which a photolabile residue positioned within the pharmacophore is allowed to form a covalent bond with a spatially-approximated residue within the receptor (35). This approach used with substitution of multiple residues spread throughout the cholecystokinin pharmacophore, providing a series of such experimentally-derived constraints (35). Receptor mutagenesis has also been utilized to gain insights into the mode of cholecystokinin docking at CCK1R and CCK2R, although this approach is indirect and is largely dependent on loss of function effects (36, 37).

It is clear that there is a small molecule-binding site in the helical bundle high in the lipid bilayer in both CCK1R and CCK2R (38, 39). This is the location of docking for benzodiazepine agonists and antagonists for these receptors. Of particular interest, structure-activity studies have localized an agonist trigger within such small molecules as an isopropyl group that interacts with receptor residue Leu7.39 (39, 40). Furthermore, the selectivity for such molecules can be changed from CCK1R to CCK2R based on the rotation at a single bond. Multiple lines of evidence have supported this site as allosteric within the CCK1R under normal conditions. Of interest, though not yet supported directly, the carboxyl-terminus of cholecystokinin has been predicted to be inserted into the helical bundle for CCK2R (37), which may occupy this region and preclude simultaneous binding of ligands here at the time peptides are bound.

To date, almost all the small molecules described to bind to CCK1R are agonists or antagonists, with minimal or no modulatory behavior for cholecystokinin action. Only one small molecule agonist has been found to also exhibit weak positive allosteric modulatory activity (41). This is perhaps not surprising, since these agents came from high throughput screening efforts directed toward this type of molecule. To date, there have been no pure positive allosteric modulators (i.e. that have no intrinsic biological activity) that have been described, which will be required to assess the utility of the proposed allosteric modulation of physiological CCK signaling.

## Impact of Cholesterol ON Cholecystokinin Receptors

Several years ago, it was recognized that cholecystokinin stimulus-activity coupling at CCK1R was abnormal at gallbladder receptors of patients with cholesterol gallstones (42–45). This was characterized by an increase in binding affinity for cholecystokinin, with reduced gallbladder contraction in response to this hormone. Indeed, those patients are also known to have bile that is super-saturated in cholesterol, with the possibility that this lipid could be transferred to adjacent cell membranes. Extensive studies have demonstrated that both CCK1R and its proximal G protein (Gq) are normal in the gallbladder muscularis of such patients, yet the coupling mechanism appears to be aberrant (42–45). This was shown to be corrected by extraction of cholesterol from such cells *in vitro*. Of note, CCK2R, a closely related GPCR, does not exhibit the cholesterol sensitivity of CCK1R. Another important control was that patients with pigment gallstones and similar inflammation exhibited normal stimulus-activity coupling at the gallbladder CCK1R. Animal models such as the prairie dog fed a high cholesterol diet confirmed this mechanism, exhibiting increased cholesterol in bile and the development of cholesterol gallstones with abnormal cholecystokinin stimulus-activity coupling present (46).

The molecular basis of the impact of cholesterol on stimulus-activity coupling at the CCK1R has been extensively explored (47, 48). Cholesterol composition of the lipid bilayer is known to affect general physicochemical characteristics, such as fluidity and the shape of the membrane. While these features were initially implicated in the impact of cholesterol on many GPCRs, it has also become clear that there can be distinct direct sites of interaction between this lipid and membrane proteins. A series of cholesterol-association amino acid sequence motifs within membrane proteins have been described, with many of these found to be present in large percentages of GPCRs. However, it is not clear how many such sites are actually utilized and how many of these are functionally important. Cholesterol association with GPCRs has been reported to affect binding affinity, signaling, and trafficking, but the impact of this has been inconsistent, with no clear rules yet established. CCK1R is one of those receptors with multiple cholesterol-association motifs in the primary sequence. Of note, CCK2R shares some of these motifs as well, despite not exhibiting the functionally important cholesterol sensitivity of CCK1R (47, 48). The cholesterol content has been modified by direct extraction and physical delivery, as well as use of drugs to affect cholesterol metabolism, and genetic modification of the cell’s machinery to produce and regulate cholesterol. The impact of cholesterol on CCK1R has been attributed to a CRAC motif low in transmembrane segment 3, with this site identified through a series of CCK1R/CCK2R chimeric constructs and then focused mutagenesis. One of the most informative CCK1R mutants involved replacement of a tyrosine residue within this motif with an alanine (49). This mimicked the effect of high cholesterol on receptor function with no further impact from increased cholesterol. The mutant receptor bound CCK and other agonists with increased affinity, while eliciting reduced intracellular calcium responses. Because there is no analogous modulation of CCK2R function by increased cholesterol, the impact of this lipid on CCK1R is clearly a direct effect.

Of further interest, sterols related to cholesterol, such as bile acids and phytosterols also potentially affect CCK1R function, adding structure-activity insights into the specificity of the cholesterol impact on this CRAC motif (50, 51). Ursodeoxycholic acid and β-sitosterol have been shown *in vitro* to reduce the negative effects of elevated cellular cholesterol on CCK1R function (50, 51).

While there has been widespread recognition of the phenomenon of cholesterol sensitivity of CCK1R in the gallbladder, it was unclear whether peripheral cholecystokinin receptors not bathed in a high cholesterol solution such as super-saturated bile would exhibit the same type of abnormality. It is thus noteworthy that there are now several reports showing the existence of abnormal membrane lipid composition in some patients who are obese or have metabolic syndrome (52, 53). This may include an increase in cholesterol, analogous to what is observed in the cholesterol gallstone patient gallbladder. Unfortunately, it is very difficult to get direct data on the characteristics of the CCK1R present on relevant enteric vagal afferent neurons. In initial efforts to evaluate the possible impact of varied membrane composition on CCK1R function, we developed techniques to rapidly express this receptor on easily accessible cells from the peripheral circulation of a broad variety of patients (54). To achieve this, we utilized adenoviral delivery of the wild type CCK1R to buffy coat cells, and quantified cholecystokinin responsiveness 24 hours later. It was impressive that there was a broad spectrum of hormonal responsiveness observed, and this correlated with various metabolic parameters such as increased body mass and diabetes. Since this was achieved *ex vivo*, it became important to explore whether the same phenomenon could be observed *in vivo*. For this, we utilized cholecystokinin-stimulated gallbladder contraction quantified using cholecystokinin-cholescintigraphy (hepatobiliary iminodiacetic acid scintigraphy, HIDA scanning) in patients having no gallbladder disease and normal full gallbladder contraction in response to hormone (55). These patients also exhibited a similar broad spectrum of cholecystokinin responsiveness. This, too, supports the presence of at least a subset of patients exhibiting reduced stimulus-activity coupling at CCK1R, with some of these patients typical of those who were likely included in previous clinical trials for the treatment of obesity. We believe that this dysfunction of cholecystokinin stimulus-activity coupling could have had a negative impact on previous trials of CCK1R agonists in obesity. Perhaps most exciting about this observation is the theoretical ability of a positive allosteric modulator to correct the dysfunctional cholecystokinin stimulus-activity coupling at this receptor. Here, too, it will be necessary to develop such agents to test this hypothesis.

## Summary

The type 1 cholecystokinin receptor continues to be a potentially important target for drugs to prevent and manage obesity, a major public health problem responsible for much morbidity and mortality. While the receptor has previously been the target of full agonists for this application, they have failed clinical trials, due to lack of superiority over acute dieting. There are substantial concerns about side effects and/or toxicity of increasing potency and duration of action of CCK1R agonists. Here, we make the case for potential advantages of biased agonists and/or positive allosteric modulators as new types of drugs targeting this receptor for obesity treatment in a safer and more effective manner. It will be important to develop such agents and to test them for proof-of-concept in a clinical setting.

## Author Contributions

All authors contributed to the article and approved the submitted version.

## Funding

This work was partially supported by a grant from the National Institutes of Health, DK115402 (LM).

## Conflict of Interest

The authors declare that the research was conducted in the absence of any commercial or financial relationships that could be construed as a potential conflict of interest.
